# Experience of Implementing a Cross-Border Primary Care Cooperation Project During the COVID-19 Pandemic: A Qualitative Study

**DOI:** 10.3390/nursrep15050178

**Published:** 2025-05-20

**Authors:** Silvia Caristia, Erica Busca, Sara Campagna, Erika Bassi, Alberto Dal Molin

**Affiliations:** 1 Department for Sustainable Development and Ecological Transition, University of Piemonte Orientale, P.zza Sant’Eusebio 5, 13100 Vercelli, Italy; silvia.caristia@uniupo.it; 2 Department of Translational Medicine, University of Piemonte Orientale, Via Solaroli 17, 28100 Novara, Italy; erika.bassi@uniupo.it (E.B.); alberto.dalmolin@uniupo.it (A.D.M.); 3 Department of Public Health and Pediatrics, University of Torino, Via Santena 5 Bis, 10126 Torino, Italy; sara.campagna@unito.it

**Keywords:** COVID-19, implementation science, family and community nursing, community health nurses, quality of health care, organizational innovation

## Abstract

**Background/Objectives**: The REACtion project was developed to provide nursing care to older adults at home within the primary care setting. The COVID-19 pandemic posed significant challenges to its implementation, acting both as a barrier and an opportunity. Although several studies have explored healthcare innovation during emergencies, there remains a need for strategic insights to guide real-world implementation efforts. This study aims to explore how the COVID-19 pandemic influenced the implementation of the REACtion project and identify the strategies adopted to ensure continuity and effectiveness in achieving its goals despite the challenges posed by the health emergency. **Methods**: A qualitative descriptive study was conducted. Semi-structured interviews were carried out with nine project stakeholders, including nurses and researchers, between April and May 2023. The interviews were transcribed verbatim, read in-depth, and analyzed using content analysis to identify perceived barriers, facilitators, and strategies adopted during the project. **Results**: The pandemic shifted priorities from routine care to emergency health activities. Barriers to project implementation included social distancing, disruptions in the decision-making processes, and a general decline in community welfare. Despite these obstacles, the COVID-19 context underscored the central role of Family and Community Nurses in proactive primary care. Stakeholders adopted relationship-based strategies, addressed workforce shortages, and implemented measures to mitigate personal fatigue. **Conclusions**: The implementation of innovative nursing interventions during emergencies requires adaptability, collaboration, and context-sensitive approaches. Strengthening stakeholder engagement and leveraging professional roles within the community are essential to overcoming barriers and seizing opportunities in crisis contexts.

## 1. Introduction

Understanding the effectiveness of project implementation requires an exploration of stakeholders’ preferences and experiences, which are crucial for identifying effective strategies, particularly in the complex field of public health interventions. Translating evidence-based interventions into practice is challenging and demands careful consideration of factors such as intervention design, local context, and behavioral strategies [1,2]. Reflection on implementation experiences enables the adaptation of plans to overcome observed barriers [1,2].

Although implementation science has garnered increasing attention in recent years, the field still lacks a unified framework for analyzing strategies, leading to considerable variability in practice [2]. To address this gap, several taxonomies have been developed, focusing on dimensions such as planning, financing, restructuring, quality management, training, stakeholder engagement, and clinical support [3,4].

These taxonomies aim to provide a more systematic approach to the adoption, sustainability, and scaling-up of innovations in healthcare settings [1,5]. However, the implementation of innovative interventions during emergencies remains an underexplored area. Emergencies are defined as sudden and unexpected situations requiring immediate action. In social science, emergencies acquire greater significance when they disrupt the social order and prompt atypical responses [6,7]. The COVID-19 pandemic exemplifies such a situation, highlighting key characteristics of social disruption as described in disaster anthropology [6].

Much of the post-pandemic literature has focused on the impact of COVID-19 and the public health measures adopted to control its spread. The pandemic reoriented healthcare priorities, shifting the organization of services toward infection control and away from the routine management of non-communicable diseases, and redirected attention from hospital-centered care to primary care settings [7]. In 2021, the World Health Organization (WHO) European Region emphasized the essential role of strong primary care systems during epidemics. These systems are critical not only for ensuring the provision of routine health services and continuity of care for individuals with chronic conditions but also for managing mild cases of SARS-CoV-2 infection [8]. The hospital-centered model—where hospitals function as the primary, and often exclusive, providers of diagnostic and therapeutic services—has limited the system’s ability to manage less urgent conditions and acute complications associated with chronic diseases [9].

As in other countries, Italy faced the pandemic within a healthcare system traditionally centered on hospital-based services, with limited integration between health and social care. During the early phases of the COVID-19 outbreak, many countries—including Italy—adopted strict containment measures, such as national lockdowns, travel restrictions, school closures, remote working, and the suspension of public gatherings [8]. These interventions were accompanied by the closure of many healthcare and public health services, particularly those related to prevention and health promotion. As a consequence, many of the primary care services, including telemedicine and mobile health technologies, were delivered remotely. General practitioners suspended in-person consultations, and most diagnostic and laboratory services were halted. There was also a notable reduction in emergency interventions for non-COVID patients. This situation led to prolonged waiting times for diagnostic exams and specialist consultations across both public and private sectors, as well as an increase in mortality among non-COVID patients [10].

The COVID-19 outbreak not only disrupted activities across various productive and economic sectors but also significantly heightened the population’s vulnerability in terms of risk for health and wellbeing, largely due to the healthcare system’s reduced capacity to manage and address routine health issues [11].

Gradually, however, services for non-COVID-related health issues reopened, and previously suspended initiatives—including those funded by the European Commission—resumed. This “return to normalcy” represents a critical phase in emergency management, even though the social and political landscape remains reshaped by the crisis. In public health, returning to normal involves ensuring the continuity of elective procedures, routine care, and community-based services. Just as the degree of service disruption varied according to countries’ health system capacity and income levels, so too did their ability to recover—largely depending on innovation capacity and the resilience of their health workforces. Key enabling factors included the rapid procurement of essential materials, scaling-up of communication strategies, redistribution of tasks, optimization of professional roles, recruitment of additional staff, and the deployment of rapid training tools and job aids [9,11,12].

This study examined the implementation of the REACtion project during the COVID-19 outbreak, interpreted as a context of social disruption. Specifically, the objectives were (i) to analyze how the pandemic influenced the implementation of the REACtion project, acting simultaneously as a barrier and a facilitator; and (ii) to identify the implementation strategies developed by stakeholders to overcome these challenges, ensure continuity in community-based primary care, and promote a new welfare model grounded in integration and resilience.

## 2. Materials and Methods

### 2.1. Design

We conducted a descriptive qualitative study using semi-structured interviews to explore the experiences and perspectives of key stakeholders involved in the REACtion project. This approach enabled an in-depth understanding of the phenomenon, allowing the researchers to derive insights from participants’ narratives and present findings in alignment with their own language and meanings [13]. The study was conducted and reported in accordance with the Consolidated Criteria for Reporting Qualitative Research (COREQ) [14].

### 2.2. REACtion Project

The REACtion project was coordinated by the University of Piemonte Orientale, in collaboration with the University of Turin, the Local Health Authorities (LHA) of Novara and Vercelli (Northern Italy), and the Locarnese and Valmaggese Association of Home Assistance and Care (ALVAD) in Switzerland. Supported by the European Regional Development Fund through the INTERREG IT-CH program, the project aimed to foster cross-border cooperation in primary care.

REACtion focused on building a healthcare network to support home-dwelling older adults through a community-based welfare model. The specific objective was to develop a joint Italian-Swiss model of care. Family and Community Nurses (FCNs) played a central role in the project, supporting older adults in maintaining health and independence at home. FCNs also provided guidance to family members and caregivers, leveraging digital technologies and facilitating access to local health and social services.

### 2.3. Participants and Data Collection

Participants included nurses, nursing directors of primary care services, and researchers actively involved in the REACtion project. Only individuals who were part of the project’s steering committee were invited to participate. Interviews were conducted between April and May 2023, either in person at participants’ workplaces or via videoconferencing, depending on availability and preference.

Interviews explored how the COVID-19 pandemic affected the implementation of the REACtion project, identifying perceived barriers and facilitators. Participants were also asked to describe the strategies adopted to address those barriers. Additional prompts encouraged participants to reflect on how their professional roles influenced the project implementation—including attitudes, behaviors, and responsibilities—and to share opinions on best practices and potential pitfalls when implementing healthcare innovations during a pandemic (Box 1).

All interviews were conducted in Italian, the participants’ native language, and lasted approximately one hour. They were carried out by a nurse researcher and a sociologist, both with extensive experience in qualitative research. During the interviews, both researchers also took field notes to capture contextual observations and immediate impressions, supporting a rich description of the data. All participants provided consent to be audio-recorded and were informed of the researchers’ roles and the study’s aims. Interviews were transcribed verbatim, anonymized, and cross-checked by two independent researchers to ensure accuracy prior to analysis.

Box 1 **Semi-structured interview used to collect information: example of questions.**

Thinking about COVID-19, what consequences did the health emergency have on the implementation of REACtion in terms of barriers or facilitators?

⁢

Thinking about those barriers, what actions were implemented to overcome them, considering the project’s limitations and resources?

### 2.4. Data Analysis

Interview data were analyzed using qualitative content analysis [15]. A coding tree was developed and applied by the two researchers. The frequency and relevance of codes, both across and within transcripts, guided the identification of emerging themes. Upon completion of coding, the researchers met to define and agree on the thematic structure in order to enhance the trustworthiness of the analysis [16]. Strategies emerging from the interviews were categorized according to the framework proposed by Kirchner et al. (2017) [5]. To further ensure the credibility and consistency of results, the data were cross-checked with minutes from the project’s monthly steering committee meetings, which included representatives from all partner institutions. Moreover, member checking was conducted with participants to validate the themes and confirm that the findings reflected their experiences.

### 2.5. Ethical Considerations

Informed consent for study participation and publication of data was obtained from participants. Researchers ensured confidentiality and anonymity through the assignment of unique alphanumeric identification codes in line with the order of recruitment. Data were returned to participants to check for accuracy and resonance with their experiences, as a technique for exploring the credibility of results. The study was conducted in full accordance with the ethical principles outlined in the Declaration of Helsinki and with current national regulations regarding research involving human participants.

## 3. Results

The study involved project coordinators and evaluators from the two Universities (N = 3), FCNs (N = 2), representatives from the LHA with managerial roles (N = 3), and one representative from the Swiss partner, responsible for coordinating activities in the Ticino Canton. Interviews lasted an average of 43 min.

The main full quotations are available in Supplementary Materials Table S1.

Five categories were identified, summarizing the main factors influencing the project’s progress. These included individual characteristics, project management, the partner (as an institutional organization), the national health system, and, broadly, the social connections within communities where the project was implemented. Each category was further analyzed according to its impact level: intra-personal, inter-personal, organizational, and policy.

### 3.1. COVID-19-Related Factors Influencing the Project

**Figure 1:**
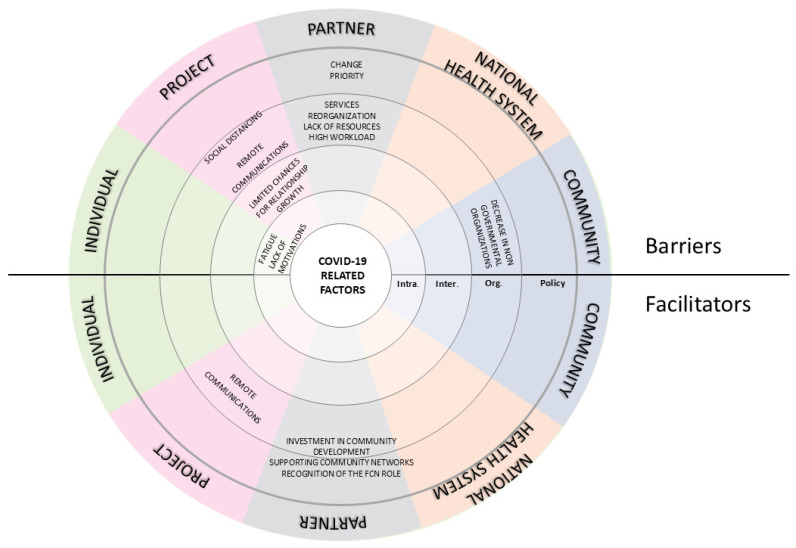
Barriers and facilitators COVID-19-related. Legends: FCN—Family and Community Nurse; Inter.—Inter-personal; Intra.—Intra-personal; Org.—Organizational.

Interviews highlighted that the COVID-19 pandemic significantly affected how the project goals were achieved. Participants described the pandemic as both a challenge and an opportunity at various levels of the system (Figure 1).

#### 3.1.1. Barriers

One of the most common barriers was the shift in institutional priorities. Managers and decision-makers had to respond to rapidly changing regulations and reallocate resources to address the health emergency, leaving little space and time available for the project. This shift slowed down project progress and restricted the time available for the active experimentation phase of REACtion. During the peak of the emergency, the project was sidelined, as stated by one participant: “*REACtion had been excluded from the Local Health Authorities (LHAs)’ internal work processes […]. COVID-19 completely inhibited the feasibility of continuing the project steps that had been defined, including their scheduled timeline*”. (S2). Another frequently reported barrier was the reallocation of healthcare professionals, which directly impacted the implementation of project activities. Nurses originally involved in REACtion were reassigned to emergency duties such as COVID-19 testing, contact tracing, and patient care in acute settings. As a result, this diversion of human resources led to increased workloads and emotional strain, particularly among managers in the project steering committee, who were responsible for ensuring the continuity of REACtion while simultaneously responding to the demands of the health crisis. Participants often viewed their involvement in the project as an added burden during the pandemic, which intensified already high workloads and emotional stress, ultimately affecting their motivation. (Figure 1). As one participant stated, “*The workload assigned to us was considerable; we enlisted the help of additional operators, but they struggled to manage the burden of both COVID-19 and REACtion. This even led us to work 10–12 h a day to fulfill both activities.*” (S3).

Within the project, COVID-19 significantly influenced internal communication methods among partners, consequently affecting inter-personal relationships and team cohesion. Remote communication replaced face-to-face meetings during lockdowns, limiting opportunities to build familiarity with the communities involved in the experimental phase. The absence of informal, in-person interactions—often essential for fostering trust and shared understanding—was perceived as a constraint to creative thinking and spontaneous problem-solving. As one participant described, “*Virtual meetings, in a way, hindered personal relationships because the video format somehow restrained that aspect. Perhaps some exchanges of ideas could have occurred more naturally in person.*” (S1). Furthermore, the heterogeneity of the partnership, involving professionals from different countries, made it necessary to adopt standardized technical terminology to ensure clarity and mutual comprehension across disciplinary and institutional boundaries (Supplementary Materials Table S1; Figure 1).

Lastly, COVID-19 led to a decline in community networks, particularly affecting support for home-dwelling older adults. In Italy especially, many associations supporting individuals with chronic illnesses ceased to operate, making it increasingly difficult for FCNs to provide care and guide citizens toward available services, as one participant stated, “*COVID-19 significantly disrupted the networks […] there was a deficiency in the development of networks aimed at supporting families.*” (S2).

#### 3.1.2. Facilitators

Although primary care integration remained limited in Italy, the pandemic unexpectedly fostered collaboration and innovation within local communities. The organization of health surveillance and care shifted towards a more collaborative model, relying on the coordinated engagement of diverse professionals and community stakeholders. This transformation was not the result of top-down planning, but rather a bottom-up response to urgent local needs, which revealed previously underutilized community resources (Figure 1). Participants described how the pandemic mobilized local resources and revealed hidden potential within the community: “*[…] The pandemic allowed us to understand the true potential of the territory, with the emergence of activities by pharmacists, […] mayors, and their networking with FCNs.*” (S2).

The emergency caused by COVID-19 also highlighted the strategic importance of effective primary healthcare, drawing increased public and institutional attention to it. In this context, the pandemic increased visibility and recognition of the FCN role, accelerating its adoption within local health systems. As one participant explained, “*In my opinion, it was COVID-19 that prompted us to implement (the FCN). […] One cannot assume a solely hospital-centered approach. The hospital should be the last option.*” (S3).

Finally, COVID-19 prompted a shift in internal communication. While physical meetings were suspended, video conferencing became a widespread tool, enabling more frequent and inclusive exchanges among project partners and supporting the continuity of coordination despite restrictions. (Supplementary Materials Table S1; Figure 1).

### 3.2. Non-COVID-19-Related Factors Influencing the Project

**Figure 2:**
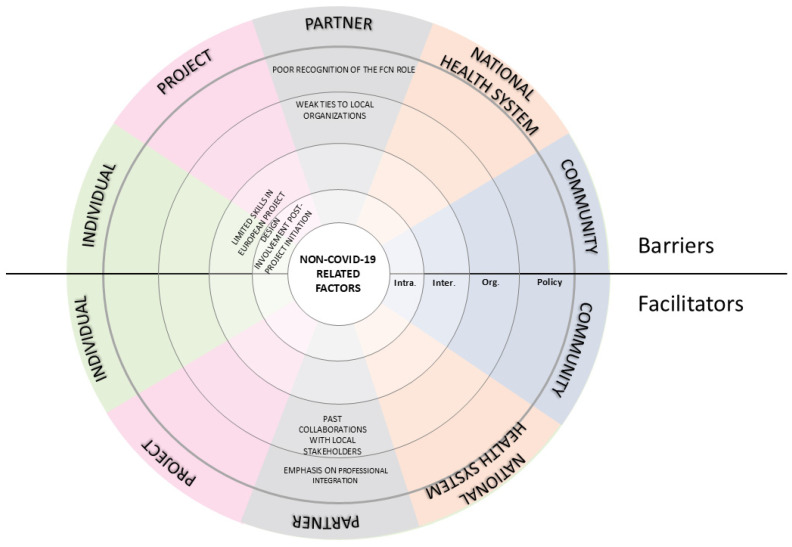
Barriers and facilitators non-COVID-19-related. Legends: FCN—Family and Community Nurse; Inter—Inter-personal; Intra.—Intra-personal; Org.—Organizational.

In addition to factors related to COVID-19, the interviews also revealed various non-COVID-19 elements that impacted the project, as illustrated in Figure 2.

#### 3.2.1. Barriers

Barriers related to the context, which existed prior to the COVID-19 pandemic, primarily concerned the LHAs in Italy. One barrier was the lack of uniformity in the introduction of the FCN role at the national level. This inconsistency generated uncertainty about the scope of practice of the role, which was often not fully recognized within the organizations (Figure 2). One participant highlighted this issue: “*The Organization did not acknowledge the work of the FCNs and, as a result, did not respond to their requests. They found themselves in a rather peculiar situation—existing but lacking significant interlocutors both within and outside the Organization.*” (S2). This lack of institutional awareness also affected the development of stable partnerships with community organizations. In some settings, collaboration remained informal and heavily dependent on individual relationships rather than structured networks. As the same participant stated, “*We directly interact with an individual within the association, someone who knows us and understands why we are reaching out. However, if that person ceases their activity within the network, we lose contact with the association*.” (S2).

Additionally, the project encountered long-standing issues in the national healthcare system, especially regarding the weak integration between hospital services, primary care, and social services. This lack of systemic coordination represented a common barrier to ensuring continuity of care and undermined the project’s ability to offer sustainable, long-term interventions for older adults. Interviews revealed that integration often occurred on a case-by-case basis, driven by individual initiative and typically involved only isolated segments of the care continuum. These collaborations were rarely formalized, and organizations struggled to establish effective interprofessional connections, which limited their capacity to respond to the complex and evolving needs of the older population. (Supplementary Materials Table S1; Figure 2).

At an intra-personal level, several stakeholders reported a lack of experience in European project management. The REACtion project was, for many participants, their first exposure to EU-funded initiatives, and required substantial efforts to navigate administrative, reporting, and technical demands. Finally, another issue concerned the timing of stakeholder involvement. Some professionals reported having joined the project only after it had already started, which made it more difficult to align with goals and activities. As one participant stated, “*If my supervisor had involved me earlier, I would have been less disoriented at the beginning. It took me some time to fully align with the project.*” (S6).

#### 3.2.2. Facilitators

The regulatory framework—especially Italy’s Ministerial Decree 77/2022—substantially supported project development by formally recognizing the role of FCNs. In addition to formally integrating FCNs into the national health system, the decree also promoted interprofessional collaboration, encouraging integration between FCNs and other health and social professionals operating in the community.

Conversely, in the Swiss context, a more advanced level of service integration was already embedded within cantonal health policies. There was a clearer orientation toward structured, community-based collaboration, which facilitated the project’s implementation. As one Swiss stakeholder explained, this collaborative model was already part of routine practice: “*With respect to the project goals, it’s something we already do—collaborating and working to strengthen the networks around the user. It’s a legal obligation for Spitex.*” (S6).

### 3.3. Implementation Strategies to Address COVID-19 Barriers

To address the barriers imposed by the COVID-19 pandemic, project partners implemented a variety of strategies across individual, partner, and organizational levels. These strategies aimed not only to adapt to the rapidly evolving health context but also to maintain engagement, restructure responsibilities, and ensure continuity in the project’s core activities. These strategies were implemented at three interconnected levels: (i) the individual level, involving personal adaptations and emotional resilience; (ii) the partner level, referring to collaboration and coordination among stakeholders; and (iii) the project level, focusing on broader project adaptations. The most frequently cited actions included strengthening stakeholder relationships, tailoring approaches to the local context, and supporting healthcare staff and nursing practice (Table 1).

#### 3.3.1. Partner and Partnership Levels

**Figure 3:**
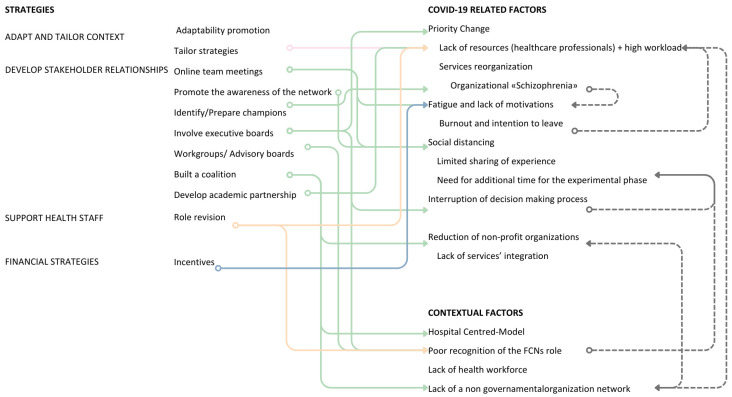
Strategies used to overcome the identified COVID-19-related barriers. Legend: The color of the arrow differs according to the type of strategy it refers to (e.g., green for DEVELOP STAKEHOLDERS’ RELATIONSHIPS). Dashed arrows highlight associations between barriers.

The shortage of professionals dedicated to non-urgent care during the pandemic was one of the most critical challenges for project implementation. The reallocation of human resources to emergency services limited the availability of staff for project-related activities, thus compromising the feasibility of the originally planned interventions. This situation prompted partners to rethink the project’s original plans, including timelines, budget allocation, and operational structure (Adapt and tailor strategies to the context) (Figure 3). Tailoring strategies primarily emerged at the partner and partnership levels, where flexibility and responsiveness became essential to navigating the changing healthcare landscape. In several cases, activities were redesigned across settings, depending on local resources and constraints. As one participant explained, “*The impact of COVID-19 was significant because the project had initially envisioned involving family nurses in a specific manner. However, due to the emergency, these plans had to be adjusted. Activities that were initially planned took on a different form, leading to a complete change in the overall planning.*” (S3).

Developing stakeholder relationship strategies was a key approach to addressing shifting priorities within the health system. These included involving executive boards in implementation efforts and engaging advisory boards and workgroups to raise awareness among LHA leadership. These strategies also aimed to address internal inconsistencies within the organization, particularly those related to the ongoing reorganization of care services, activity planning, and staff responsibilities. In response to operational fragmentation, some partners identified and prepared “champions” (i.e., individuals with strong motivation and strategic positioning, who were entrusted with promoting and sustaining key implementation tasks). The executive board was also involved in addressing the interruption of decision-making processes related to “ordinary” health issues (Figure 3). Their participation provided formal legitimacy to the project and contributed to realigning institutional priorities with the evolving needs of community-based care. This strategic engagement also served to increase the visibility and recognition of the FCN role. As one participant recalled, “*We aimed to make [the top management] understand our initiatives. We achieved this by actively involving them in events, making sure they saw what was really happening. The breakthrough came during our first event […] From then on, whenever possible, either [the director] or a representative attended our events. This demonstrated their recognition of the added value our initiatives brought.*” (S3).

Monthly online team meetings were introduced to support personal motivation and alleviate work overload, while also preserving relationships despite social distancing policies (Figure 3). These virtual meetings created a space for ongoing dialogue and coordination, compensating for the loss of in-person interaction. As one participant noted, “*Perhaps the most difficult thing, but also the most important, was to maintain contact with all partners, even during the most difficult phases of the pandemic. […] I remember numerous phone calls with partners to stimulate and reignite their motivation for this project.*” (S1).

One partner reported building a coalition with local stakeholders to address the weakening of Non-Governmental Organizations (NGOs), formally establishing a partnership between LHA and local volunteer organizations. Another partner described the creation of an internal coalition within the organization, while a third highlighted the formation of an academic partnership to help address shortages of human and financial resources (Table 1).

To facilitate project activities and support health staff, some partners revised the allocation of project tasks, reallocating responsibilities among involved actors to better balance workloads during the experimental phase (Figure 3). This process often required individuals in coordination roles to make pragmatic decisions about delegation and resource redistribution. As one participant explained, “*I said ‘I have to delegate if I want to do it!’, because otherwise I wouldn’t have been able to carry on within the LHA. […]*” (S3).

Finally, only one partner adopted financial strategies, modifying incentive and allowance structures to formally recognize the overtime work carried out by FCNs, thereby boosting workforce motivation.

#### 3.3.2. Individual Level

At the individual level, strategies focused on reinforcing personal engagement, initiative, and resilience in a context of high uncertainty. Two key approaches emerged: (i) the identification and support of internal “champions” to guide implementation efforts, and (ii) the promotion of relationship-building dynamics (network weaving) to maintain motivation and ensure continuity (Figure 3). Some members of the steering committee, holding managerial roles within their organizations, contributed to this process by actively encouraging collaboration among colleagues—often through personal initiative. As one manager described, “*I had to move independently and seek collaboration, step by step, with several people.*” (S3).

Some of these strategies, although developed in response to the COVID-19 emergency, were also described by participants as effective in addressing broader, non-COVID-related barriers. In particular, involving executive boards and promoting network weaving were considered applicable beyond the emergency context and potentially beneficial for routine project implementation. For instance, the poor recognition of the FCN role was mitigated by engaging decision-makers through executive-level involvement. Similarly, limited integration between health services and local organizations was addressed by fostering interprofessional connections through network weaving.

### 3.4. Lessons Learned

The REACtion project provided important insights into the management of collaborative processes and team dynamics, including (a) establishing a coordination team to meet deadlines and guide project execution; (b) formalizing an internal working group to reinforce accountability and professional commitment; (c) adopting flexible working modalities; and (d) fostering collaboration across institutions (Supplementary Materials Table S1).

Participants emphasized the importance of aligning perspectives and building a shared understanding of project goals within the LHAs. They recommended addressing this issue through a preliminary feasibility assessment during the proposal development phase. As one stakeholder reflected, “*Before implementing a project, it’s important to assess the feasibility, considering human, structural, and technological resources. Having these resources is essential for project success […] Although COVID wasn’t a factor when we proposed the project, no one considered how it might impact our timeline.*” (S3).

#### Post-Pandemic Reflections

Although participants were not directly asked about the sustainability of the REACtion project beyond the COVID-19 pandemic, interview analysis revealed perspectives on aspects that remained relevant in the post-emergency phase. The findings suggest that the pandemic highlighted the strategic value of investing in primary healthcare and strengthened recognition of the proactive role played by FCNs, particularly in supporting older adults and vulnerable populations. The COVID-19 pandemic created a favourable context for accelerating the implementation of primary care models and strengthening collaboration between health services and informal community networks. These dynamics were aligned with the principles underlying the REACtion project. As one participant observed, “*The pandemic made partners reflect even more on the importance of investing in primary healthcare, and therefore in family and community nursing. But not only that—it also helped to fully understand that primary care cannot stand alone, but must go hand in hand with community involvement. […] In my opinion, although it was never explicitly stated, the pandemic led the partners to give concrete form to this idea: the importance of formal and informal networks that could, in some way, offer support.*” (S1).

Two key aspects of the project appeared to have continued in the post-pandemic phase: (a) stronger interconnection with NGOs, such as local volunteer associations, which supported the development of generative welfare models based on community social support; and (b) greater awareness of the need to strengthen the presence of FCNs across the territory, in line with efforts to enhance the primary care system and promote a more integrated model of care. As one participant stated, “*Healthcare management became aware that, in the end, Family and Community Nurses needed to be increased and expanded.*” (S2).

## 4. Discussion

The REACtion project was designed to strengthen the care network supporting frailty among older people, through a model of proximity and integrated health and social care. The COVID-19 pandemic exacerbated existing vulnerabilities and exposed the structural limitations of the healthcare system—particularly those associated with a predominantly hospital-centered model of care. During the pandemic, this hospital-based approach proved inadequate in managing non-COVID patients, contributing to increased mortality in this population group. This awareness led to a paradigm shift in many LHAs, promoting policies aimed at community-based and integrated care models, as recommended by the WHO European Region. In this context, the FCN role emerged as a liaison among health, social services, and NGOs. The REACtion project effectively aligned with this shift, demonstrating its potential to respond to systemic healthcare challenges. Building on this experience, the present study analyzes the barriers and facilitators encountered during the implementation of the REACtion project—a European public health initiative—within the context of the COVID-19 pandemic. It further explores the strategies adopted by stakeholders to ensure its integration into an evolving and complex healthcare landscape.

Among the barriers encountered during the implementation of the REACtion project, particularly in the phase affected by the COVID-19 pandemic, several critical challenges emerged that adversely impacted the continuity and effectiveness of healthcare services. These included the redirection of attention from non-emergency to emergency care, the effects of social distancing measures, disruptions in local decision-making processes, and the reduced operational capacity of local NGOs. Additionally, fatigue and demotivation among project partners further hindered the execution of planned interventions. The pandemic also profoundly challenged the organization of care services, exposing latent structural tensions that hindered effective coordination and timely responses. As this study reveals, three recurring governance challenges played a critical role in shaping the implementation and adaptation of care strategies: breakdowns in information flow, unclear or contested authority, and weak inter-organizational coordination [17,18]. These issues were consistently reflected in interviews with project stakeholders, highlighting how systemic inefficiencies and fragmented governance mechanisms complicated service delivery and delayed collaborative decision-making.

Despite these constraints, the pandemic also served as a catalyst for innovation and adaptive strategies. Within this challenging context, stakeholders mobilized internal resources to maintain project continuity and redefine healthcare delivery models. The crisis fostered new forms of collaboration, enhanced the visibility of key professional roles (e.g, FCNs), and revealed previously underutilized assets within community networks.

In response to the identified barriers, a series of implementation strategies was developed and applied to ensure the project’s continuity and alignment with evolving care needs. Although tailored to specific local contexts, these strategies converged around four core areas: (a) building stakeholder relationships to address shifting priorities; (b) coping with the shortage of skilled workforces; (c) mitigating the weakening of community welfare networks; (d) sustaining engagement and motivation among members of the project steering committee.

These strategies align with the principles outlined in the WHO–UNICEF Operational Framework for Primary Health Care (PHC) (2020), which builds on the vision of the Declaration of Astana (2018) [19,20]. The framework emphasizes the importance of coordinated, multisectoral action to strengthen PHC and drive health system transformation. Central to this vision is investment in a well-trained, equitably distributed primary care workforce. In this context, adaptability, the development of integrated care networks, and targeted financial support emerged as essential not only for managing the acute and recovery phases of the COVID-19 pandemic but also for addressing the needs of vulnerable populations, particularly those with chronic conditions, such as cardiovascular disease. This is especially relevant considering that cardiovascular disease has been recognized not only as a comorbidity that worsens COVID-19 outcomes, but also as a potential sequela of SARS-CoV-2 infection itself [21].

The use of tools for remote communication, including telemedicine and teleconsultation, became a common strategy among national health systems to manage non-communicable diseases during the pandemic. Several studies—from both high- and low-income countries—have reported the widespread implementation of these approaches, alongside financial and political efforts to support the integration of services for chronic disease management [22,23]. At the same time, social distancing policies disrupted collaboration and interprofessional relationships. Although virtual meetings ensured operational continuity, they often lacked the emotional nuance and spontaneity of in-person interactions, potentially hindering focus, teamwork, and innovation [18,24]. Another strategic response was the reinforcement of community-based care, which recognizes the vital role of informal caregivers, such as family members and neighborhood networks. Strengthening formal and informal networks was seen as essential for improving population wellbeing and quality of life [25,26]. This perspective is supported by both national and international experiences, including Italian initiatives aimed at supporting informal caregivers of older adults with dementia [27], as well as the Burlington Ontario Health Team’s model for managing care in patients with chronic conditions [28]. In an effort to strengthen care networks, the REACtion project placed a central emphasis on the role of FCNs. These professionals were key in integrating formal and informal care systems by fostering connections between healthcare services, NGOs, and informal caregivers to address both the explicit and latent health needs of the population, particularly within vulnerable groups. Comparable experiences have been documented internationally—for instance, in Thailand, where community nurses were instrumental in supporting older adults through the promotion of self-care practices, ultimately reducing their susceptibility to SARS-CoV-2 infection [29,30].

The final strategic area identified in this analysis concerns the challenge of sustaining engagement and motivation among healthcare professionals, especially in the context of prolonged crisis and post-emergency recovery. Literature widely documents the psychological toll of the SARS-CoV-2 pandemic on health workers [31,32,33]: professionals involved in the care of COVID-19 patients experienced elevated levels of stress, burnout, secondary traumatic stress, anxiety, and depression. These issues were exacerbated in regions with higher infection rates, where staff reported reduced compassion satisfaction. Within this context, motivation emerged as a key variable influencing the level of engagement in new initiatives, particularly those not directly related to COVID-19. A lack of motivation was identified as a significant barrier to participation, especially during the recovery phase. However, findings from this study—and consistent with broader literature—highlight that supportive work environments and a strong sense of perceived organizational support can act as powerful enablers of motivation and sustained engagement [34,35].

A key limitation of this study was the absence of perspectives from patients and their informal caregivers, who represent the primary beneficiaries of the REACtion project interventions. Instead, the study relied on insights from healthcare professionals, including both frontline staff and decision-makers within the involved LHAs. While this approach enabled the identification of shared concerns and recurring themes across different organizational levels, it may have limited the understanding of how the REACtion project effectively permeated care pathways and influenced the care experience from the perspective of its beneficiaries.

## 5. Conclusions

The implementation strategies observed in this study—contextual adaptation, stakeholder engagement, and workforce support—are well-established in non-emergency settings [5] and proved equally effective during a crisis. The REACtion project, through innovative practices such as FCN integration and proactive care models, contributed to a broader healthcare transition toward community-based systems. This aligns with a broader global shift in healthcare systems, including the trend towards de-hospitalization and the integration of health services with community care networks to support care transition [36,37,38,39].

While the qualitative nature of the research and the small number of participants may restrict the generalizability of the findings, this study offers a deep understanding of the implementation process and action strategies to mitigate the impact of COVID-19, as confirmed by the literature. Peters et al. highlighted the significance of prioritizing strategies that facilitate effective intervention implementation, particularly in emergencies like the pandemic [40]. Moreover, this is not the only study that has attempted to observe the implementation strategies of an innovative public health intervention in a pandemic context [22,23,27].

Several key lessons emerge from this experience that may inform future health emergency preparedness. First, participants emphasized the value of multisectoral collaboration and the formalization of working groups, as well as the importance of fostering structured relationships among different institutional and community stakeholders. Second, role flexibility, extended timelines, and adaptive planning were seen as essential enablers for modifying interventions in response to the evolving pandemic scenario and context-specific needs. Third, the presence of a shared vision and clearly defined common goals across organizations and communities was regarded as fundamental for effective action. Equally important was the presence of strong and supportive leadership. Leaders who demonstrated the ability to coordinate teams, sustain motivation, and act as bridges between frontline realities and institutional priorities were instrumental in ensuring continuity and cohesion during project implementation. These elements are strongly supported by existing literature and should guide the development of institutional frameworks for health system resilience [41,42,43].

From a clinical practice perspective, this experience also reinforces the need to invest in the primary care workforce. In particular, the role of FCNs emerged as a crucial connector to improving continuity of care, especially for patients with chronic conditions or reduced autonomy. Strengthening this role within multidisciplinary teams may be key to building more resilient and person-centered health systems.

Given these findings, future studies should prioritize quantitative methodologies to assess the effectiveness of these types of policy interventions on health and wellbeing outcomes. Currently, much of the available evidence derives from case studies or context-specific reports, which, while rich in detail, often lack statistical power and generalizability. Longitudinal designs that follow cohorts of populations exposed to similar healthcare service models—compared to unexposed groups—could yield critical insights to inform evidence-based public health policy at both local and national levels.

## Figures and Tables

**Table 1:** Strategies applied according to the level at which they operated.

	Level of Actions						
	Individual	Partner		Project			
	Participants (N)	Participants (N)	Partners (N)	Participants (N)	Partners (N)	Total Participants	Total Partners
Development stakeholder relationships							
Build a coalition				2	2		2
Develop academic partnership				1	1	1	1
Identify and prepare champions	1	2	1			3	1
Involve executive boards				2	2	2	2
Online implementation team meetings				1	1	1	1
Promote network weaving	2	1	1	2	2	4	3
Use advisory boards and workgroups				2	2	2	2
Adapt and tailor to context							
Promote adaptability				4	3	4	3
Tailor strategies		2	2	1	1	3	3
Support clinicians							
Revise professional roles	1			2	2	2	2
Utilize financial strategies							
Alter incentive/allowance structures		1	1			1	1

## Data Availability

The datasets generated during and/or analyzed during the current study are available from the corresponding author on reasonable request.

## Supplementary Materials

The following supporting information can be downloaded at: https://www.mdpi.com/article/10.3390/nursrep15050178/s1, Table S1: Main full quotations.

## Public Involvement Statement

No public involvement in any aspect of this research.

## Guidelines and Standards Statement

This manuscript was drafted against the Consolidated Criteria for Reporting Qualitative Research (COREQ) for qualitative research.

## Abbreviations

The following abbreviations are used in this manuscript:

FCN	Family Community Nurse
LHA	Local Health Authority
NGO	Non-Governmental Organization
