# Supplementary material for: Experience of Implementing a Cross-Border Primary Care Cooperation Project During the COVID-19 Pandemic: A Qualitative Study
Source: Nurs Rep. 2025 May 20;15(5):178. doi: 10.3390/nursrep15050178 (PMC12114520; doi:10.3390/nursrep15050178)
Supplement: Supplementary file 1 [file nursrep-15-00178-s001.zip › nursrep-3582517-supplementary.pdf]

SUPPLEMENTARY MATERIALS

Table S1: Main full quotations

**COVID-19**	Participants (N)	Main full quotations
Barriers		
Change priority	6	"Certainly, the time available (for the project's activities) was reduced due to momentary needs, necessitating prioritization. We had to navigate what I would call 'organizational schizophrenia,' as rules kept changing, requiring a constant reassessment of our organizational approach. [...] time constraints were a reality. I won't hide the fact that we expressed our difficulties, as we were genuinely facing significant challenges." (S8)<br><br>"REACtion had been excluded from the Local Health Authorities (LHAs)' internal work processes, e.g. all FCNs were called to manage the families of the exposed by telephone (contact tracing) by mandate of the LHA [...] there was no longer any kind of proactive and project exchange; COVID-19 completely inhibited the feasibility of continuing the project steps that had been defined, even temporally (S2)."
Services reorganization	3	"REACtion had been excluded from the Local Health Authorities (LHAs)' internal work processes, e.g. all FCNs were called to manage the families of the exposed by telephone (contact tracing) by mandate of the LHA [...] there was no longer any kind of proactive and project exchange; COVID-19 completely inhibited the feasibility of continuing the project steps that had been defined, even temporally (S2)."<br><br>"We had to let go of the individuals we were responsible for - I'm sorry to say this - because COVID inundated us with a multitude of other tasks. Additionally, it was a decision from the Directorate-General: 'Set aside what you were doing before; there are new priorities now' (S7)."
Lack of resources	6	"So, the pandemic undeniably impacted the project. Initially, the impact seems negative, as certain operational partners responsible for implementing experimental interventions—such as health structures—were heavily engaged in addressing the pandemic emergency. For instance, some nurses dedicated to territorial care were reassigned to perform swabs or even to care for patients in the acute phase, sometimes within hospitals in COVID wards. Not only nurses but also the leaders, those overseeing various groups, found themselves shouldering new responsibilities. I recall one member of the project, particularly affected due to her role in the ASL, struggling to attend meetings as she was consistently called back by management or other services to address

		the emergency. This constant demand (by LHA) left little room for engagement with the REACtion project, making it challenging for us to have meaningful dialogues and discussions." (S1)
High workload	2	"The workload assigned to us was considerable; we enlisted the help of additional operators, but they struggled to manage the burden of both COVID-19 and REACtion. This even led us to work 10-12 hours a day to fulfill both activities" (S3).
Social distancing - remote communications<br><br>Limited changes for relationship growth	3<br><br>2	"The inability to meet in person was a challenge for me. I won't say it created problems, but it took a while for me to adapt to the project and carry out all the activities remotely. In the initial phase, there might have been a lack of concreteness, but for obvious reasons—it was challenging to kick-start things. [...] As the project progressed, however, even though the physical absence hindered our initial acquaintance and limited time spent together, all aspects were thoroughly discussed during steering committee meetings. The schedule was adhered to, thanks to effective coordination. I believe it would have been different if all meetings were conducted in person, allowing more participation in the organized events. That certainly would be better." (S6)<br><br>"In a quieter setting, we could have accomplished more. For instance, having the opportunity to move around, to visit the locations where we performed the experimental phase (of the project)—essentially, being more physically present—would have made a significant difference. Yes, the moments we were physically present allowed for more socialization and experiences beyond the project. Virtual meetings, in a way, hindered personal relationships because the video format somehow restrained that aspect. Perhaps some exchanges of ideas could have occurred more naturally in person—during dinner, while out for a walk, or when casually comparing notes. These informal settings tend to foster the emergence of ideas, unlike in a formal meeting. This is perhaps another drawback of relying on technology in meetings; it has increased efficiency but may have compromised some of its effectiveness." (S1)
Fatigue<br><br>Lack of motivation	2	"The workload assigned to us was considerable; we enlisted the help of additional operators, but they struggled to manage the burden of both COVID-19 and REACtion. This even led us to work 10-12 hours a day to fulfill both activities [...] [Referring to the fact that the first people involved in REACtion had left] It was a highly stressful situation; they experienced burnout, and there was a moment when I felt in crisis too because I didn't know where to turn anymore. It meant continuing to cover shifts, opening activities, and reorganizing. On the other hand, the project coordination was setting deadlines for us [laughing]. In the end, we managed to pull through, and we are also happy." (S3)

		“All the partners, despite facing difficulties, fears, and a desire to halt the project, continued to work (for the project). Perhaps they didn't express it verbally... I recall some challenging meeting where the piloting was temporarily halted. In these instances, various criticalities and difficulties emerged, leaving me devastated due to the sense of responsibility I felt for advancing the project. However, it turned out that the partners themselves had already devised strategies to overcome the significant challenges that had arisen during the meeting the day before [...] During the pandemic, I also found myself involved in extra activities related to the pandemic." (S1)
Non governmental organization reduced	1	"It was a unique impact that I observed, requiring a dedicated effort to comprehend the situation. COVID-19 significantly disrupted the networks, as activities with volunteers, shopkeepers, and key figures like general practitioners, parish priests, and mayors in the *** area had all come to a standstill. There was a deficiency in the development of networks aimed at supporting families."(S2)
Facilitators		
Remote communications	3	“We all worked remotely, and paradoxically, during remote work, the opportunities for meetings were more frequent than when working in person. Strangely, when normal activities resumed, in-person meetings and other opportunities for contact were perhaps a bit less frequent.”(S4)
Investment in territorial development and supporting community networks	3	"The associations that survived COVID-19 became highly active and played a crucial role in providing support to all citizens. […] The pandemic allowed us to understand the true potential of the territory, with the emergence of activities by pharmacists, […] mayors, and their networking with FCNs”(S2)<br><br>“Before the COVID-19 pandemic, establishing contacts on the ground was more challenging. For instance, when reaching out to the mayor, one had to adhere to the hierarchies of the organizational structure (LHA). However, during COVID-19, everything became possible and easier [...] (S7)
Recognition of the FCN role	3	“The management (of LHA) became aware that there should be plans to increase and disseminate family and community nurses." (S2)<br><br>"In my opinion, it was COVID-19 that prompted us to implement (the FCN). Without COVID-19, progress would have been much slower. [...] The turning point came with Ministerial Decree 71 and then 77. It was from there that the understanding emerged—capillary presence of FCNs on the territory is crucial. One cannot assume a solely hospital-centered approach. The hospital should be the last option where the patient must arrive” (S3)

**CONTEXTUAL FACTORS**		
Barriers		
Poor recognition of the FCN role	2	"The Organization did not acknowledge the work of the FCNs and, as a result, did not respond to their requests. They found themselves in a rather peculiar situation - existing but lacking significant interlocutors both within and outside the Organization" (S2).
Weak ties to local organizations	1	"The district has always operated with a reactive mechanism and has never counted on formal networks of volunteers, etc. The first time the district engaged with pharmacists, for instance, was through the national-level involvement with the 'farmacia amica' project, and currently, only one location, ***, is actively participating in it. [...] The FCNs perceived a certain fragility in relations (with associations). In fact, they expressed: 'We directly interact with an individual within the association, someone who knows us and understands why we are reaching out. However, if that person ceases their activity within the network, we lose contact with the association.'"(S2)
Limited skills in European design	5	"[In reference to the factors impacting the experimental phase] This is the first project I'm participating in, and enrolling for the use of the device was not easy. Many people refused for various reasons, ranging from 'I don't wear a watch' to 'I don't want to be monitored,' and some simply did not want to commit. [...] Despite the challenges, I believe the concept of the device is quite interesting. Getting this device accepted has, in my opinion, been not so easy." (S5)<br><br>"I have faced challenges, but I am uncertain about how much they are directly related to the pandemic. Apart from the University ***, which has research offices with a structured staff, the other project beneficiaries lacked the necessary resources. They had never been involved in a European project before, and suddenly, they found themselves managing one and having to report their expenses. I recall the first year when we had meetings practically every week with the Front Office. These meetings were crucial for understanding what a European project entails, the process of handling expenses, and discerning between eligible and unallowable expenses—essentially, navigating through all the project documentation." (S9)
Involvement post-project initiation	2	"If I had been involved at an earlier stage (in the project), I would have been less lost initially. It took me a moment to align with the project" (S6)
Facilitators		
Past collaborations with the territory	2	"Twice a year, the LHA conducts meetings with local associations. This is a regular event where the LHA introduces what it can offer, and the associations, in turn, present themselves and their projects. So, for the main associations, we already established contacts." (S3)

		“(At the table with the associations) we initially participated as auditors and then we sought involvement in the projects they had initiated, realizing that they might address our needs [...] (as a result) we established connections with social workers who also attended the meetings (S8)."
Emphasis on the integration of professionals	3	“(At the table with the associations) we initially participated as auditors and then we sought involvement in the projects they had initiated, realizing that they might address our needs [...] (as a result) we established connections with social workers who also attended the meetings (S8)."<br>“With respect to the project goals, it's something we already do—collaborating and working to strengthen the networks around the user. It's a legal obligation for Spitex." (S6)
**IMPLEMENTATION STRATEGIES TO ADDRESS COVID-19 BARRIERS**		
Developing of stakeholders’ relationship		
Build a coalition	2	**partner level:**<br>"Because we involved those (FCNs) from the northern area (of the District) in the project, even if they were not officially part of it. When we organized events, we always invited them, and they always attended. When interventions were carried out in the VCO, we always tried to send someone, because the project was relevant to the entire organization" (S3)<br><br>“[Regarding the cooperation agreement] We maintain a valuable collaboration with the associations in Santhià. This collaboration remains crucial because, without them, many of our families wouldn't be able to fully engage in the care process. When you're facing financial challenges and can't afford transportation services, which I can cover... this becomes a crucial support. This collaboration started during a specific period, gained strength with the cooperation agreement, and has been consistently ongoing.”(S7)
Develop academic partnership	1	**partner level:**<br>[Interviewer: One of the approaches you adopted to fulfill the project's objectives was also recruiting resources that were not initially planned but that you fortunately managed to incorporate into the project.] Let's say that *** [external resource obtained through collaboration with the University] was a pleasant surprise at that time. Thanks to contacts with *** [university professor], we were able to identify *** [new resource] as the most suitable candidate to fulfill this project requirement. Very few nurses were actually involved... *** [university professor] has been collaborating with *** [partner] for years as an external consultant. This is probably one of the facilitating factors that allowed this collaboration to continue, even when *** [LHA] took a step back. (S6)
Identify and prepare champions	3	**individual level:**

		“I had to move independently and seek collaboration, step by step, with several people”(S3)<br><br>**partner level:**<br>"When *** [REACtion coordinator] gave us the timelines, saying 'we need to do this thing within this period,' we already had our deadlines, and then *** [REACtion manager in the Local Health Authority] would define who would do what” (S8)
Involve executive boards	2	**partner level:**<br>"To help top management comprehend the role of family and community nurses and persuade them to sign the Santhià Charter [an agreement between the Local Health Authority (LHA) and nonprofit organizations in the area] was quite a challenge [said with a laugh]. For them, it was more of a political matter, whereas for the family and community nurses, establishing networks was a substantial concern” (S2)<br><br>"[Regarding the management of the Local Health Authority (LHA)]. We aimed to make them understand our initiatives. We achieved this by actively involving them in events, making sure they saw what was really happening. The breakthrough came during our first event on Viale Roma for the International Day of Cooperation. Although we had briefed the top management on our plans, the real understanding dawned on them as they toured the stands. From then on, whenever possible, either he or a representative attended our events. This demonstrated their recognition of the added value our initiatives brought.” (S3)
Online implementation team meetings	1	**project level:** "So, let's say that the COVID-19 pandemic has inevitably halted some projects, right? Essentially, we had to rethink the steering meetings,originally planned to be on-site. At a certain point, we had to consider holding them remotely. But I must say that, in hindsight, it was an opportunity. The pandemic forced us to find new strategies, for example, to manage remote meetings, which we now find indispensable. So much so that the other day, when we said, “Shall we have the next meeting for the fourth call in person?” Perhaps we'll see, as you've noticed, doing them remotely works well. Sometimes the meetings are even more organized [...] it ensures greater attendance and so on." (S1)
Promote network weaving	4	**individual level:** “during the pandemic, physical and mental presence at some point was taken away because they were physically and mentally disturbed by some huge stuff that was happening. My effort was to maintain a steady course because this was crucial. Therefore, I listened to everyone, seeking to leverage all weaknesses and critical issues reported by partners [...] there there were moments in which the situation was difficult to carry forward [...] partners raised significant concerns that couldn't be ignored [...] however I must say that perhaps the most difficult thing, but also the most important, was to maintain contact with all partners, even

		during the most difficult phases of the pandemic, we always hold the meetings [...] If someone couldn't participate, it was understood, and perhaps there was a phone call in the evening to provide information or to motivate. I remember numerous phone calls with partners to stimulate and reignite their motivation for this project.” (S1)<br><br>**partner level:** “[Regarding local associations] during COVID some reinvented themselves, while others didn't. This is why we took the initiative as promoter and we're continuing to do so”(S3)<br><br>**project level:** "There was a sharing; it was one of the strengths of the project, and the regular meetings were a condition that did not create rivalry but always led to cohesion [emphasizing cohesion as it is said], with a significant capacity for support even from individual partners”. (S4)<br><br>“Always create this sort of team dynamic because, in my opinion, personal dynamics are what help you achieve results. If you share the dynamics and the goal, it's easy for motivation to be strong.” (S8)
Use advisory boards and workgroups	2	**partner level:**<br>"So, several times, tables were set up with the district management so they (FCNs) could present a snapshot of what they had observed in families. [...] So, initially, there was a dialogue with the district to make the top management understand the role of family and community nurses.”(S2)<br><br>"We have conducted multiple project presentations at separate sessions for the health director, administrative director, and general director, aiming to illustrate..." (S3)
Adapt and tailor to context		
Promote adaptability	4	**project level:**<br>“There have definitely been changes, adopting great flexibility in timing, resizing some of the objectives [...] we have learned to try to rationalize the work as much as possible because we truly realized the inhumane workload [emphasizing it] that the people working in the territory and anyone working in a LHA had. The project essentially added to activities that were already being carried out. Therefore, the principle of parsimony in data collection and requests“ (S4)<br><br>“Coming to terms with reality. Nurses can do this at the moment; they can have this availability of time and can meet in these ways. Therefore, we have adjusted based on what we had envisioned, taking into account the needs of the beneficiary, and we have communicated this to the funder." (S9)

Tailor strategies	3	**partner level**: "We requested the nurses to resume control of the mapping process and start anew. They initiated a fresh analysis, consistently referring to the district's printouts to identify the families and, indeed, the survivors"(S2)<br><br>"We tried to rationalize resources as much as possible, meaning we aimed to activate them precisely where it was strictly necessary, without affecting the operational efficiency of the service because [...] being a public interest entity, we cannot avoid taking on a request. Therefore, we must try to activate ourselves as effectively as possible."(S6)<br><br>**project level:**<br>"[Referring to COVID-19] The impact of COVID-19 was significant because the project had initially envisioned involving family nurses in a specific manner. However, due to the emergency, these plans had to be adjusted. Activities that were initially planned took on a different form, leading to a complete change in the overall planning. Initially, there was a notion of implementing the same technological and network intervention across all territories [...] However, due to the COVID-19 emergency, the areas of focus shifted. For example, *** took a different direction, prompting us to reconsider our approach, focusing on addressing falls." (S3)
Support health staff		
Revise professional roles	2	**individual / project level:**<br>[referring to the excessive work commitment in managing the pandemic and REACtion] I said "I have to delegate if I want to do it!", because otherwise I wouldn't have been able to do it in the LHA. I wouldn't have secured the contract, I wouldn't have succeeded and so *** [another partner] took over, specifically managing the platform. In that sense, now the network is progressing forward anyway" (S3)<br><br>**project level:**<br>"Yes, I have to say that at times, we moved beyond our roles as evaluators to provide additional support for the project. Therefore, we worked closely with others [...] Quite often, our role resembled more of a partner than an evaluator, aiming to support the project's progress.I'll give you an example, theoretically the platform and the production of manuals on how to purchase a device were not within our purview. But on the other hand I must also say that the partners did evaluate, handle, and advance certain aspects of the evaluation, which were more closely related to our role." (S4)
Utilize financial strategies		
Alter incentive/allowance structures	1	**partner level:**

		"So, we did a lot of work involving FCNs, and it was strongly based on the premise of, 'Look, you have to do it for me once your service hours are over.' I couldn't allow them to skip their shifts for this other activity [...] All the hours worked outside were recognized. Therefore, everything they did beyond their regular duties was accommodated within their service hours. This was the only leverage, so to speak [laughing], that I had because I didn't have anything else. I always tried to motivate them as much as possible. This is one thing that entices people even more. It's one thing to be as motivated as I could be or as someone else was, while someone who understood that they wouldn't receive extra left the project."(S3)
